# Experimental study on heat transfer characteristics of plate evaporator under heaving, pitching, and rolling conditions

**DOI:** 10.1038/s41598-025-31723-6

**Published:** 2025-12-10

**Authors:** Hongju Chen, Bangting Yu, Yiping Zhang, Yuhang Zhou, Cui Li, Yonghu Wu, Xu He, Dan Hua, Chengbin Zhang

**Affiliations:** 1 CNOOC China Limited, Beijing Research Center, Beijing, 100028 China; 2 Key Laboratory of Efficient Low-Carbon Energy Conversion and Utilization of Jiangsu Provincial Higher Education Institutions, Suzhou University of Science and Technology, Suzhou, 215009 Jiangsu China; 3 School of Energy and Environment, Southeast University, Nanjing, Jiangsu 210096 People’s Republic of China

**Keywords:** Heaving, Pitching, Rolling, Plate evaporator, Flow boiling, Heat transfer correlation, Engineering, Renewable energy

## Abstract

To investigate the variation characteristics of heat transfer performance in plate evaporators within the ocean thermal energy conversion (OTEC) systems of underwater unmanned vehicles (UUVs) under marine motion conditions, a flow boiling experimental system integrated with a six-degree-of-freedom motion platform was designed and established. This study examined the effects of sloshing modes (heaving, pitching, and rolling), mass velocity, sloshing amplitude, sloshing frequency, and sloshing intensity on the heat transfer characteristics of the plate evaporator. The results indicate the following: (1) Heaving motion exerts a significant enhancement effect on the heat transfer performance of the plate evaporator. Under conditions of a heaving amplitude of 100 mm and a frequency of 0.6 Hz, the convective heat transfer coefficient of R134a increases by up to 61.8%; (2) Pitching motion exhibits a noticeable enhancement effect on heat transfer performance at small sloshing amplitudes (2.5° amplitude), with the convective heat transfer coefficient of R134a increasing by up to 34.75% at a frequency of 0.2 Hz; (3) Rolling motion demonstrates a significant weakening effect on heat transfer performance, with the convective heat transfer coefficient of R134a decreasing by up to 31.8% under conditions of a rolling amplitude of 7.5° and a sloshing frequency of 1 Hz. It should be noted that the inlet working fluid of the plate evaporator in this experiment is saturated, and the vapor quality of the outlet working fluid is 0.3–0.4. Furthermore, a heat transfer correlation applicable to plate evaporators under heaving, pitching, and rolling conditions was developed in this study. Validation results demonstrate that the proposed correlation exhibits excellent predictive performance, with prediction deviations within ± 15%. It should be noted that the applicable range of the heat transfer correlation established in this study is for pitching and rolling amplitudes of 2.5° to 7.5°, heaving amplitudes of 50 to 100 mm, frequencies of 0 to 1 Hz, mass fluxes of 125 to 225 kg·m-2·s-1, and the working fluid being R134a.

**Supplementary Information:**

The online version contains supplementary material available at 10.1038/s41598-025-31723-6.

## Introduction

**Fig. 1:**
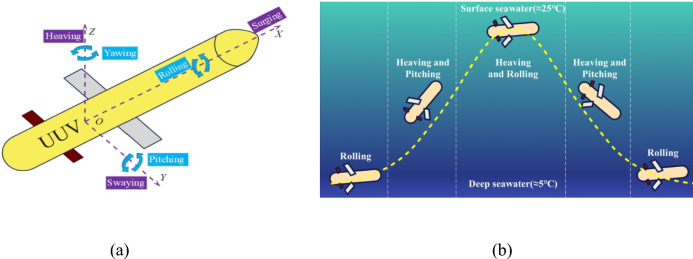
The motion form of UUVs in the ocean.

Underwater Unmanned Vehicles (UUVs) play a crucial role in ocean exploration^1–3^. However, their operational range and endurance are limited by the finite battery capacity^4^. Ocean energy (including tidal energy, wave energy, ocean current energy, and ocean thermal energy) is a renewable resource with abundant reserves^5^. Compared to chemical batteries, renewable energy sources can provide sustained electric power to UUVs, thereby significantly enhancing their operational range and sampling capability. Additionally, using renewable energy can substantially reduce the environmental pollution caused by chemical batteries. Therefore, renewable energy is widely regarded as the primary energy supply source for the next generation of UUVs^6–9^. The most common method for harnessing ocean thermal energy is ocean thermal energy conversion (OTEC)^10–13^. Plate evaporators are extensively used in these OTEC systems. Compared to other types of heat exchangers, plate evaporators offer advantages such as large heat transfer area, high efficiency, compact structure, high structural strength, and minimal end temperature difference^14^. UUVs experience six degrees of freedom (DOF) motion when navigating the ocean, as illustrated in Fig. 1(a). These six DOF motions include heaving, swaying, surging, rolling, pitching, and yawing^15^. To achieve ocean thermal energy conversion, UUVs need to travel between surface seawater and deep seawater, absorbing thermal energy at 25 °C and 5 °C. During this process, UUVs are also influenced by ocean currents and underwater obstacles. Therefore, the primary motions of UUVs in water are heaving, pitching, and rolling, as shown in Fig. 1(b). The additional inertial forces generated by these motions cause uneven distribution of gas–liquid phases within the plate evaporator^16–18^, leading to changes in heat transfer characteristics and increased flow instability^19,20^. Currently, the heat transfer performance of plate evaporators under marine motion conditions has not been fully studied. This paper investigates the effects of different motion forms (heaving, pitching, and rolling) on the heat transfer characteristics of R134a plate evaporators, aiming to provide a reference for the feasibility of using plate evaporators in UUV energy supply systems.

At present, the study of heat transfer characteristics of plate heat exchangers mainly adopts experimental methods. Walraven et al.^21^ experimentally analyzed the single-phase and boiling heat transfer characteristics of three brazed plate heat exchangers in the organic Rankine cycle and obtained an empirical formula for the evaporation heat transfer of the working fluid R245fa in the brazed plate heat exchanger, with an average absolute error of 9.97%. Huang et al.^22^ conducted heat transfer and pressure drop experiments on R134a and R507A in three different industrial application plate heat exchangers. The results showed that when the outlet vapor quality of the plate heat exchanger was 0.2 ~ 1, the boiling process was dominated by nucleate boiling. The main reason was that the change in the heat transfer coefficient was mainly affected by the heat flux density and less by the mass flow flux. Khan et al.^23^ conducted heat transfer and pressure drop experiments of ammonia-water in a plate heat exchanger. The experimental results showed that the saturation temperature had a significant impact on the heat transfer coefficient, and both the heat transfer coefficient and pressure drop increased with the increase in saturation temperature. Lee^24^ experimentally studied the relationship between the flow boiling heat transfer coefficient of R134a under low mass flow conditions and vapor quality, heat flux, evaporation pressure, and mass flow flux. The results showed that nucleate boiling was predominant in the plate heat exchanger, and the mass flow flux had a weak effect on the heat transfer coefficient. Han et al.^25^ experimentally studied the heat transfer and pressure drop performance of R410A and R22 in a plate heat exchanger, exploring the effects of mass flow flux, saturation temperature, vapor quality, heat flux density, and plate geometry on heat transfer. The flow boiling behavior of working fluids in heat exchanger channels should focus on multiphase flow dynamics in confined environments. Most studies adopt combined numerical simulation and experimental techniques to clarify flow mechanisms, aiming to optimize flow control and improve prediction accuracy. Chen et al.^26^ develops a phase-field multiphase lattice Boltzmann model to investigate droplet dynamics in microfluidic T-junctions, innovatively revealing that lubricating film formation and critical vortex strength determine non-breakup. Chen et al.^27^ numerically investigates double emulsion droplet dynamics in shear flow, revealing inner droplets form uniform vortical flow under steady deformation, increasing integral resistance at high volume fractions. This study provided theoretical support for multiphase flow control. Li et al.^28^ addresses two-phase flow challenges in confined spaces: vapor backflow and chaotic patterns deteriorating thermal transport. A thermal regulator integrating Tesla valves and capillary structures is developed, eliminating backflow and promoting ordered liquid flow. It switches states to enhance heat transfer coefficient and critical heat flux. Liu et al.^29^ combines visualization experiments and DPIV to investigate electrohydrodynamic deformation mechanisms of droplets in combined electric field-shear flow, clarifying surface charge convection’s enhancement/suppression under different R/S, and developing a modified model to improve *ϕ*_*d*_ prediction accuracy, supporting microfluidic droplet manipulation and multiphysics flow research.

For heat transfer characteristics under marine motion conditions, existing studies mainly cover single-phase flow within channels and two-phase flow in spiral wound heat exchangers. Dong et al.^30^ investigated the impact of sinusoidal rotational motion on the velocity and pressure fields inside a spiral wound heat exchanger through numerical simulation, finding that sloshing led to periodic changes in the velocity and pressure fields, with reciprocating motion between the tube and shell enhancing fluid mixing. Zheng et al.^31^ studied the effect of uneven distribution of gas–liquid mixtures on the heat transfer characteristics of plate fin heat exchangers (PFHEs). The results showed that under experimental conditions, with the increase in sloshing amplitude, the inhomogeneity of the two-phase mixture increased by 1.25 ~ 18.03% compared to the steady state and decreased with the increase in sloshing period. Under mixed sloshing conditions, the increase range of two-phase inhomogeneity was 14.8 ~ 27.9%. Du et al.^32^ numerically investigated the heat and mass transfer characteristics of gas–liquid two-phase mixed refrigerants on the shell side of a SWHE, finding that sloshing enhanced heat exchange performance, although sometimes the effect was not significant. Sloshing helped to reduce flow resistance. The overall performance of multi-component refrigerants was improved, with more significant improvements under sloshing conditions. Ren et al.^33^ numerically studied the flow and heat transfer characteristics on the shell side of a SWHE under pitching conditions, finding significant and periodic impacts. The effects of pitching and heaving were more pronounced than rolling, and the impact of sloshing frequency was smaller than that of sloshing amplitude. Additionally, the regularity of fluctuations in two-phase flow and heat transfer characteristics decreased with the increase in vapor quality. Different heat exchanger structural parameters had varying impacts on heat transfer performance under sloshing conditions. Hu et al.^34^ investigated the flow boiling characteristics in the finned channels of plate fin heat exchangers under different sloshing conditions, including rolling, pitching, and swaying. The results indicated that rotational types of motion could increase the instantaneous heat transfer coefficient by up to 21.1% at low vapor quality, while decreasing it by up to 27.7% at high vapor quality. Hu^35^ also studied the impact of sloshing conditions with zigzag channels on the performance of printed circuit heat exchangers (PCHEs). The results showed that rolling and heaving had significant impacts on performance. Rolling conditions always deteriorated flow boiling heat transfer in PCHEs, with a maximum reduction of 14.0%; this deterioration mainly occurred in annular flow and intensified with increasing vapor quality due to uneven lateral gas–liquid distribution at the channel inlet. Heaving conditions always enhanced flow boiling heat transfer in PCHEs, with a maximum enhancement of 22.8%, corresponding to the critical point of flow regime transition from slug flow to annular flow. Zhou^36^ studied the impact of sloshing conditions on condensation heat transfer in metal foam-wrapped tubes, finding that rolling significantly affected condensation heat transfer, while the other five forms of sloshing had negligible impacts.

Motion conditions significantly impact the heat transfer performance of heat exchangers, with the degree of influence depending on factors such as heat exchanger structure type, motion form, amplitude, and frequency. However, current research on heat transfer characteristics under motion conditions remains in the preliminary exploration stage, with existing achievements primarily confined to numerical simulations, particularly lacking experimental studies on plate evaporators under dynamic conditions. This study innovatively established a six-degree-of-freedom motion simulation experimental platform, conducting systematic experimental investigations on the heat transfer performance of plate evaporators under heaving, pitching, and rolling motion conditions. It aims to reveal the influence mechanisms of different motion parameters on heat transfer laws.

## Experimental apparatus and data processing

### Experimental setup

**Fig. 2:**
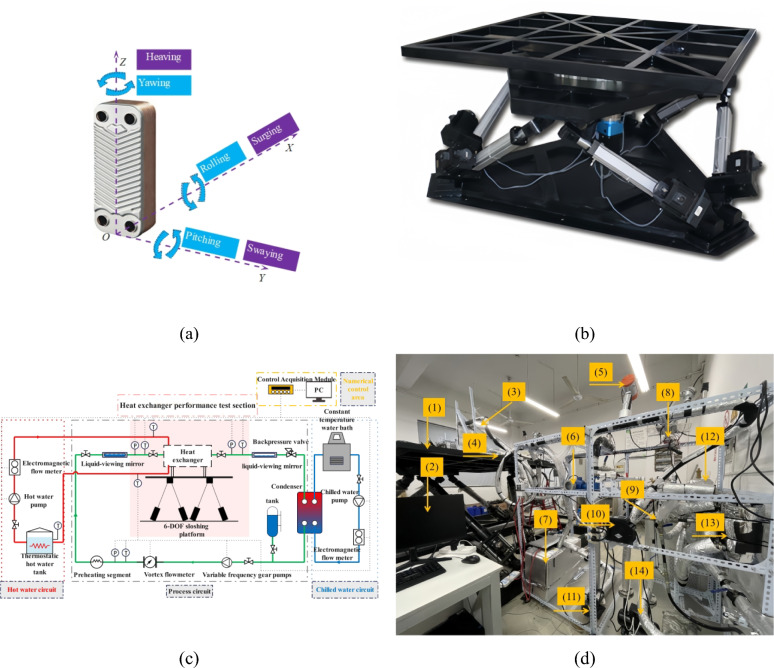
Heat exchanger test platform under sloshing conditions (**a**) Plate Heat exchanger (**b**) 6-DOF motion platform (**c**) Experimental system. All test elements and equipment in (**d**) are shown below: (1) 6-DOF motion platform (2) Computer (3) Plate heat exchanger (4) Preheating segment (5) Vortex flow meter (6) Electromagnetic flow meter (7) Hot water tank (8) Data acquisition module (9) Cold water source (10) Inverter (11) Water pump (12) Working fluid storage tank (13) Condenser (14) Gear pump.

The motion of Unmanned Underwater Vehicles (UUVs) in marine environments can be decomposed into six degrees of freedom: three translational (surge, sway, heave) and three rotational (pitch, yaw, roll) motions. For ocean thermal energy conversion, UUVs require thermal energy absorption from the seabed (~ 5 °C) and sea surface (~ 25 °C), necessitating vertical navigation approximating simple harmonic motion (Fig. 1(b)). Distinct motion characteristics are observed during operational phases: (1) seabed cruising predominantly involves rolling movements for obstacle avoidance; (2)vertical transit (seabed-to-surface/surface-to-seabed) exhibits heave-pitch dominance with bow-up/bow-down attitudes; and (3) surface gliding, influenced by ocean currents, primarily manifests heave and roll motions. This study utilized a 6-DOF motion platform (Fig. 2(b)) to simulate heaving (50/75/100 mm amplitude), pitching (2.5°/5.0°/7.5°), and rolling (2.5°/5.0°/7.5°) motions across 0–1 Hz frequency intervals (0.2 Hz increments), as specified in Table 1. It should be noted that the stable condition corresponds to the 0 Hz condition in the figure, that is, at 0 Hz, the sloshing platform is in a static state, and the system is in a stable state without interference from external forces.

**Table 1:** Designed experimental conditions for motion.

Condition	Direction	Amplification	Frequency Range	Precision
Heaving	Z Translation	50 mm/75 mm/100 mm	0 ~ 1 Hz	0.5 mm
Pitching	XZ Rotation	2.5°/7.5°/7.5°	0 ~ 1 Hz	0.1°
Rolling	YZ Rotation	2.5°/7.5°/7.5°	0 ~ 1 Hz	0.1°

A plate evaporator (Fig. 2(a)) was selected as the experimental subject, with 316 stainless steel as the plate material. Detailed structural parameters of the plates are provided in Table 2. The experimental system for evaluating heat transfer performance was designed and constructed following the methodology described by Fei^37^, consisting of three integrated loops: working fluid, cold water, and hot water circuits (Fig. 2(c)). The operational sequence was as follows: working fluid was pumped from the storage tank through a gear pump into the preheating section, entering the evaporator in a saturated state. Within the evaporator, heat absorption from the hot water circuit induced phase change to a two-phase state. Post-evaporation, the working fluid passed through a pressure reducing valve before entering the condenser, where heat exchange with cold water facilitated condensation back to liquid phase, completing the cycle (Fig. 2(d)). The experimental conditions in this study are set as follows: the mass flux of hot water is 175 kg·m^−2^·s^−1^, with a temperature of 25 °C; the cold water temperature is 5 °C; the mass flux of the working fluid are 125 kg·m^−2^·s^−1^, 175 kg·m^−2^·s^−1^, and 225 kg·m^−2^·s^−1^, with the plate evaporator inlet temperature set at 10 °C. It should be noted that the inlet working fluid of the plate evaporator in this experiment is saturated, and the vapor quality of the outlet working fluid is 0.3–0.4. All system components were thermally insulated to minimize ambient heat loss.

**Table 2:** Structural parameters of plates.

Structural parameters	Value
Length of plate/(mm)	340
Width of plate/(mm)	185
Average width between panels/(mm)	2.6
Thickness of plates/(mm)	0.6
Number of plates	10
Heat exchange area/(m^2^)	0.063
corrugation angle/(β)	30°

The origin O is located at the center of the bottom of the plate heat exchanger(as shown in Fig. 2(a).The x-axis is perpendicular to the heat exchange plates; the y-axis is parallel to the wide side of the heat exchange plates; and the z-axis is parallel to the long side of the heat exchange plates. In addition, it is specified that the inclination angle θ of the plate heat exchanger in the vertical posture is 0°, the clockwise rotation direction around the axis is the positive direction, and the counterclockwise rotation direction around the axis is the negative direction.

### Data reduction

The principle of thermal equilibrium is the theoretical basis for calculating the heat transfer coefficient of heat exchangers: the thermal equilibrium between the hot and cold fluids, i.e., the absorbed heat and the released heat are basically equal. The cross-sectional area is denoted as S, which refers to the flow cross-sectional area of the flow channel. The calculation formula is S = N × w × d (where N is the number of flow channels, w is the width of the flow channel, and d is the plate spacing). Therefore, the time-averaged total heat transfer coefficient is defined by Eqs. (1)-(3). Equation (4) is the formula for calculating the convective heat transfer coefficient.

1 $$U=\frac{Q}{A\cdot {\Delta T}_{m}}$$

2 $$Q={q}_{m,water}{c}_{p,water}({T}_{hot,\mathrm{w}ater,in}-{T}_{hot,\mathrm{w}ater,out})$$

where q_*m,water*_ refers to the mass flow rate of water, with the unit of kg/s. In Eq. (2), to achieve ocean thermal energy conversion, UUVs need to travel between surface seawater and deep seawater, absorbing thermal energy at 25℃ and 5℃. Therefore, the set temperatures of water in the experiment were 25℃ and 5℃ respectively. It should be clarified that "*T*_*hot,water,in*_" in Eq. (2) refers to the inlet temperature on the water side of the plate evaporator. To simulate the operating conditions of the UUV in marine environments, its temperature is set to 25℃. "*T*_*hot,water,out*_" in Eq. (2) refers to the outlet temperature on the water side of the plate evaporator.

The LMTD (Log Mean Temperature Difference, *ΔT*_*m*_) is a key parameter in plate heat exchanger design. For counter flow arrangement (where the hot and cold fluids flow in opposite directions), the formulation is:

3 $${\Delta T}_{m}=\frac{{\Delta T}_{max}-{\Delta T}_{min}}{\mathrm{ln}(\frac{{\Delta T}_{max}}{{\Delta T}_{min}})}$$

In most refrigeration and energy applications involving water and refrigerant, counterflow is preferred for higher thermal efficiency. Thus, the water and refrigerant sides typically use a counterflow configuration. *ΔT*_max_: Maximum temperature difference at one end of the heat exchanger (e.g., for counterflow: hot fluid inlet-cold fluid outlet); *ΔT*_*min*_: Minimum temperature difference at the other end (e.g., for counterflow: hot fluid outlet-cold fluid inlet).

4 $${h}_{r,sat}=\frac{1}{\frac{1}{{U}_{sat}}-\frac{\delta }{{\lambda }_{pl}}-\frac{1}{{h}_{water,sat}}}$$

In Eq. (4), the value of the plate material (316 stainless steel) at operating temperature 25℃ (16.2 (W·m^−1^·K^−1^)) was used. The heat transfer coefficient of water in the plate evaporator is obtained based on the modified Wilson plot method. For the specific process, refer to Appendix A.

### Sloshing intensity

**Fig. 3:**
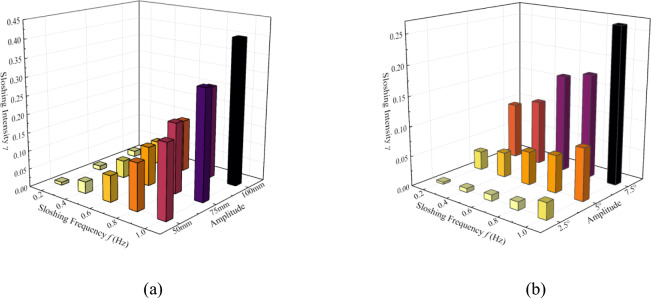
(**a**) Values of the sloshing intensity under translation motion (**b**) Values of the sloshing intensity under rotational motion.

Sloshing intensity (γ) was defined as the ratio of sloshing acceleration (*a*_*sl*_) to gravitational acceleration (with *g* taken as 9.8 m·s^−2^) to quantify sloshing severity. Under translational heaving motion (Fig. 3(a)), γ exhibited a approximately linear relationship with both amplitude (50/75/100 mm) and frequency (0–1 Hz). For rotational motions (pitching/rolling, Fig. 3(b)), amplitude (2.5°/5.0°/7.5°) exerted a more significant influence on γ than frequency, with maximum γ values observed at the highest amplitude. While γ increased with frequency at constant amplitude, this effect was quantitatively weaker than amplitude dependence. Notably, frequency-amplitude coupling induced complex additional inertial forces, which may perturbation flow boiling characteristics of the working fluid within the plate evaporator. The six-degree-of-freedom sloshing platform is directly measured by a built-in three-axis acceleration sensor (range ± 2 g, sampling rate 1 kHz). The time-domain signal is converted into acceleration power spectral density (PSD) through Fourier transform, and the root mean square acceleration is obtained by integration. The calculated value is directly displayed on the computer.

### Uncertainties

In this experiment, both temperature parameters and flow parameters are directly measured by instruments. Due to the limited accuracy of the experimental measurement points, there will be direct measurement errors during the experiment. Moreover, parameters such as the convective heat transfer coefficient *h*_*sl*​_ and the total heat transfer coefficient *U*, which cannot be directly measured, also have indirect measurement errors. These errors are functions of the directly measured errors. According to the principle of error propagation, the indirect measurement error can be calculated. Assuming the indirectly measured quantity *y* = *f* (*x*_*1*_, *x*_*2*_, *x*_*3*_​), where *x*_*1*_​, *x*_*2*_, *x*_*3*_ are independent directly measured quantities, each directly measured with equal precision multiple times and only containing random errors, the measurement error of the indirectly measured quantity *y* can be calculated using Eq. (5):

5 $${\sigma }_{y}=\sqrt{{\left(\frac{\partial f}{\partial {x}_{1}}\right)}^{2}{\sigma }_{{x}_{1}}^{2}+{\left(\frac{\partial f}{\partial {x}_{2}}\right)}^{2}{\sigma }_{{x}_{2}}^{2}+\cdots +{\left(\frac{\partial f}{\partial {x}_{n}}\right)}^{2}{\sigma }_{{x}_{n}}^{2}}$$

The uncertainties of the experimental instruments and calculated parameters are shown in Table 3. The maximum relative uncertainty of the convective heat transfer coefficient of the working fluid is ± 3.4%

**Table 3:** Uncertainties in experimental instrumentation and calculated parameters.

Instrument uncertainty	Typology	Measurement range	Uncertainties
Temperature sensor	PT100	−50 ~ 150℃	± 0.15℃
Pressure sensors	Diffusion Silicon Manometer	0 ~ 2500 kPa	0.5%FS
Flowmeter	Electromagnetic flow meter	0 ~ 3m^3^/h	0.5%FS
	Vortex flow meter	0 ~ 3m^3^/h	0.5%FS

It should be noted that the percentages in the table represent absolute errors based on full scale.

## Experimental results and discussion

### Influence of heaving motion

#### Influence of heaving motion on convective heat transfer performance

The experimental results of convective heat transfer in the plate evaporator under heaving motion (simulating UUV operations) demonstrate that the convective heat transfer coefficient (HTC) of R134a exhibits a consistent trend with sloshing frequency across different mass fluxes (125, 175, 225 kg·m⁻^2^·s⁻^1^): increasing to a peak before decreasing, yet remaining above steady-state levels even at 1 Hz. The degree of HTC fluctuation intensifies with both heaving amplitude and mass flux. Peak enhancements were observed under specific conditions: 8.12% and 32.67% at 50 mm amplitude, 0.4 Hz, and 125/175 kg·m⁻^2^·s⁻^1^; 19.21–49.98% at 75 mm, 0.6 Hz; and 23.17–61.8% at 100 mm (125 kg·m⁻^2^·s⁻^1^ at 0.4 Hz, 175/225 kg·m⁻^2^·s⁻^1^ at 0.6 Hz). Notably, the optimal heat transfer condition occurs at 100 mm amplitude, 0.6 Hz frequency, and 175 kg·m⁻^2^·s⁻^1^. Enhanced heat transfer in the low-to-mid frequency range (0–0.6 Hz) is attributed to additional inertial forces from heaving, which intensify flow boiling by promoting turbulence thereby accelerating liquid film evaporation and bubble dynamics. However, excessive frequency (> 0.6 Hz) leads to premature termination of the boiling process due to overly vigorous inertial forces, causing uneven gas–liquid distribution and diminished heat transfer efficiency. The observed phenomena arise from the interplay between heaving-induced inertial forces and two-phase flow dynamics. At low-to-moderate frequencies, periodic acceleration/deceleration of the evaporator creates oscillatory flow perturbations, enhancing convective mixing and increasing the interfacial area between the refrigerant and heat transfer surface—key factors for boosting HTC. Conversely, high-frequency heaving generates excessive centrifugal forces, leading to stratified or slug flow regimes, which reduce effective heat transfer area and induce local dry-out spots. The mass flux dependence further indicates that inertial forces interact synergistically with shear stress: higher mass fluxes amplify flow perturbations at lower amplitudes, while optimal coupling between heaving intensity (γ) and mass flux (175 kg·m⁻^2^·s⁻^1^) balances disturbance intensity and flow stability, maximizing convective heat transfer through enhanced turbulent mixing.

#### Influence of heaving acceleration on heat transfer performance

**Fig. 4:**
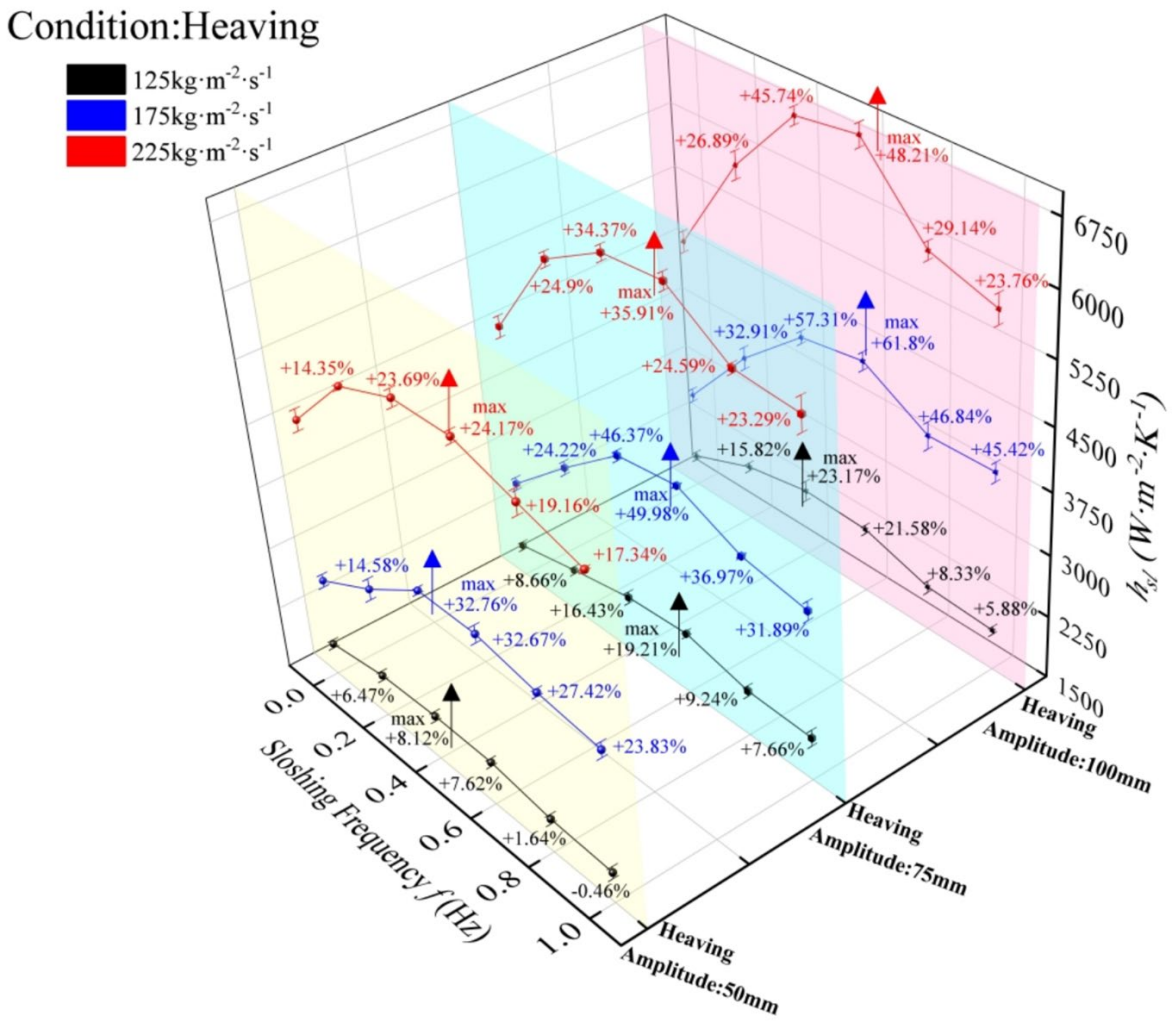
Convective heat transfer coefficient of R134a under heaving motion.

To address the operational challenge of measuring sloshing amplitude and frequency in UUVs, this study characterizes sloshing severity using the dimensionless intensity parameter γ = aₛₗ/g (g = 9.8 m·s⁻^2^) and evaluates convective heat transfer performance via the Nusselt number Nu (Fig. 4), revealing that heaving motion significantly enhances plate evaporator heat transfer compared to stable conditions (γ = 0). Among them, *a*_*sl*_ represents the sloshing acceleration, which is used to uniformly express the coupling effect of different sloshing amplitudes, sloshing angles, and sloshing frequencies on the heat exchanger. For mass flux of 175 and 225 kg·m⁻^2^·s⁻^1^, sloshing intensity emerges as the dominant factor, with Nu exhibiting distinct regimes: significant enhancement under low γ (0–0.1), oscillatory behavior with peak enhancement at γ = 0.15 (61.8% and 48.21%, respectively) in the medium range (0.1–0.2), and stabilization under high γ (0.2–0.4). In contrast, at 125 kg·m⁻^2^·s⁻^1^, mass flux dominates, leading to gradual Nu increase in low γ (0–0.1), damped oscillations in medium γ (0.1–0.2), and stabilization in high γ (0.2–0.4) with reduced oscillation amplitude. Mechanistically, low γ enhancement arises from the superposition of heaving-induced additional forces and axial inertial forces, accelerating liquid film rupture and bubble nucleation, while high γ attenuation results from excessive oscillatory forces disrupting gas–liquid distribution; optimal heat transfer enhancement (61.8%) occurs at γ = 0.145 and 175 kg·m⁻^2^·s⁻^1^, balancing inertial perturbation and phase distribution stability. Low-to-moderate sloshing intensities (γ ≤ 0.2) generate periodic flow perturbations that enhance turbulent mixing and increase refrigerant-wall interfacial area—key factors amplifying nucleate boiling and bubble dynamics. At optimal γ = 0.145, the synergistic coupling of inertial forces and mass flux (175 kg·m⁻^2^·s⁻^1^) balances flow disturbance and stability, maximizing convective heat transfer. Excessive sloshing intensity (> 0.2) introduces dominant centrifuga forces, triggering stratified regimes that reduce effective heat transfer area and induce local dry-out, while insufficient mass flux (125 kg·m⁻^2^·s⁻^1^) limits shear stress, diminishing the disruptive effect of inertial forces on boundary layers. Controlled perturbations promote nucleate boiling, whereas excessive instability disrupts phase distribution and boiling continuity.

### Influence of heaving motion on the total heat transfer coefficient

**Fig. 5:**
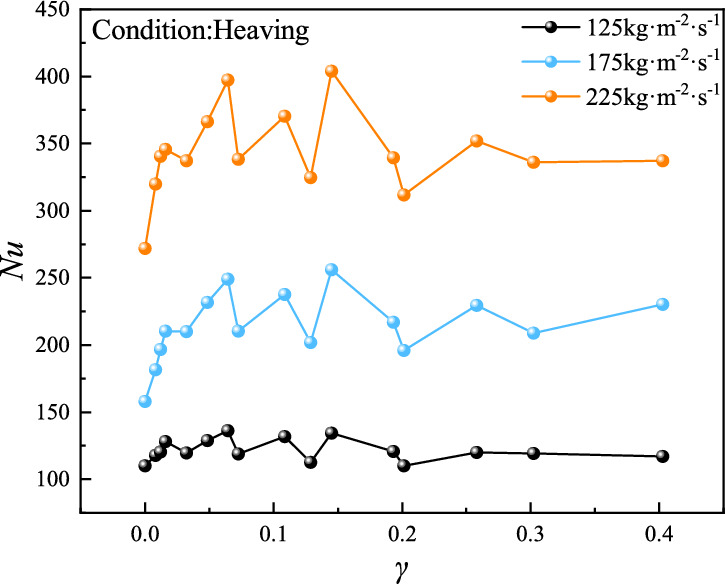
The variation of heat transfer performance with sloshing intensity under heaving motion.

**Fig. 6:**
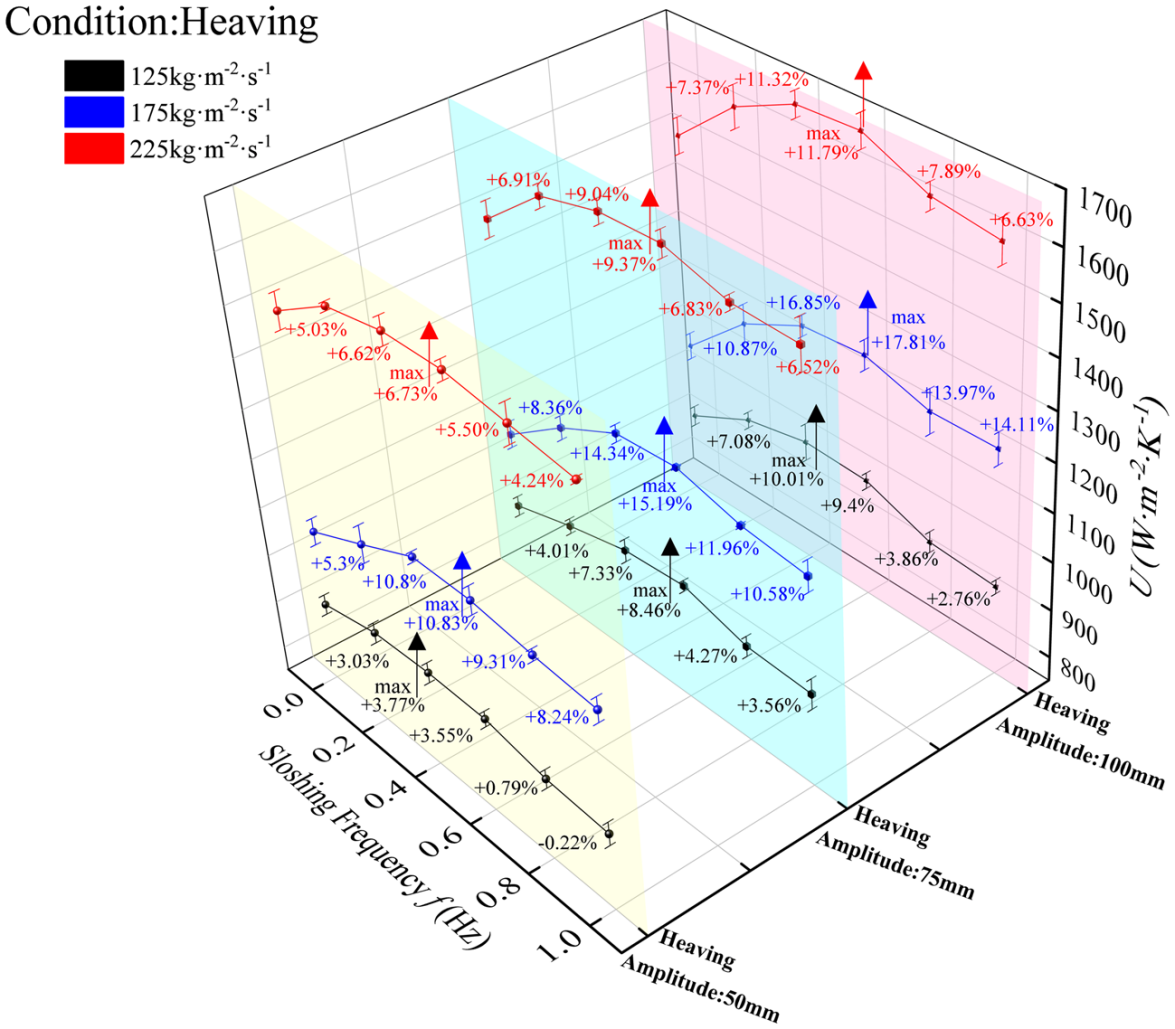
Total heat transfer coefficient of plate heat exchanger under heaving motion.

The total heat transfer coefficient (HTC) of the plate evaporator, a key performance metric for UUV thermal energy conversion systems, exhibits frequency-dependent behavior in Fig. 5 consistent with Fig. 6, with all heaving conditions outperforming stable operation (0 Hz) even at minimum HTC values observed at 1 Hz across amplitudes (50 mm, 75 mm, 100 mm). At 1 Hz, 50 mm amplitude yielded marginal enhancements: 125 kg·m⁻^2^·s⁻^1^ (comparable to static), 175 kg·m⁻^2^·s⁻^1^ (8.24%), and 225 kg·m⁻^2^·s⁻^1^ (4.24%); 75 mm amplitude showed moderate improvements: 3.56%, 10.58%, and 6.52% for the same flux; while 100 mm amplitude produced the highest gains at 175 kg·m⁻^2^·s⁻^1^ (14.11%) alongside 2.76% (125 kg·m⁻^2^·s⁻^1^) and 6.63% (225 kg·m⁻^2^·s⁻^1^). Across amplitudes, frequency effects followed a unified trend: significant HTC enhancement under stable (0–0.2 Hz) and harsh (0.2–0.5 Hz) motion regimes, with partial attenuation in extreme frequencies (0.5–1 Hz) where mass flux emerged as the dominant factor governing heat transfer performance. At low-to-moderate frequencies (0–0.5 Hz), periodic inertial forces generate controlled flow perturbations that enhance turbulent mixing, and increase refrigerant-wall interfacial area—mechanisms that amplify nucleate boiling and bubble detachment, particularly pronounced at higher mass fluxes (175–225 kg·m⁻^2^·s⁻^1^) where shear stress synergizes with inertial forces to intensify boundary layer disruption. As frequency approaches 1 Hz, excessive inertial forces induce stratified regimes, reducing effective heat transfer area and triggering partial dry-out. The superior performance at 175 kg·m⁻^2^·s⁻^1^ (e.g., 17.81% enhancement at 100 mm/0.4 Hz) reflects optimal coupling between inertial perturbation intensity and mass flux-driven shear stress, balancing flow disturbance and stability to maximize convective heat transfer. Conversely, lower mass flux (125 kg·m⁻^2^·s⁻^1^) limits shear-induced boundary layer disruption, diminishing the amplifying effect of inertial forces and resulting in marginal enhancements even at extreme frequencies.

### Influence of pitching motion

#### Influence of pitching motion on convective heat transfer performance

**Fig. 7:**
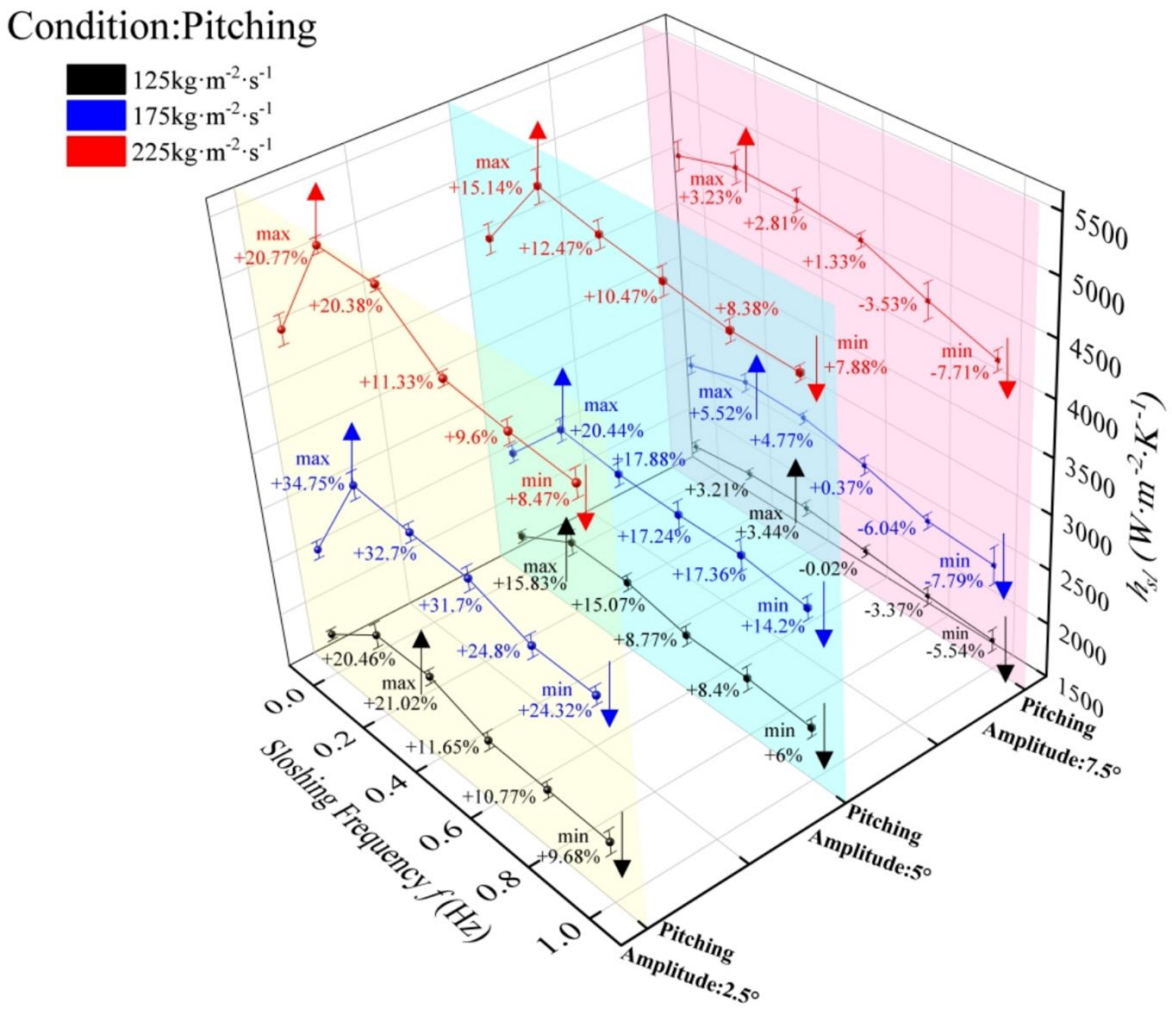
Convective heat transfer coefficient of R134a under pitching motion.

The experimental results of convective heat transfer in the plate evaporator under simulated UUV pitching motion (Fig. 7) demonstrate consistent trends across mass flux: the convective heat transfer coefficient (HTC) increases with pitching frequency to a peak, then decreases, reaching a minimum at 1 Hz for R134a. At constant mass flux, HTC fluctuation magnitude and enhancement efficiency both decreased with increasing pitching amplitude, with heat transfer deterioration occurring at 7.5°. For 2.5° amplitude, HTC peaked at 0.2 Hz for 175/225 kg·m⁻^2^·s⁻^1^ (34.75%/20.77% enhancement) and at 0.4 Hz for 125 kg·m⁻^2^·s⁻^1^ (21.02%); at 5° amplitude, peak HTC occurred uniformly at 0.2 Hz (15.83%, 20.44%, 15.14% for 125/175/225 kg·m⁻^2^·s⁻^1^); at 7.5° amplitude, marginal peaks were observed at 0.2 Hz for 175/225 kg·m⁻^2^·s⁻^1^ (5.52%/3.23%), while 125 kg·m⁻^2^·s⁻^1^ peaked at 0.4 Hz (3.44%), with deterioration onset at 0.8 Hz and minimum HTC at 1 Hz (−7.79%/−7.71% for 175/225 kg·m⁻^2^·s⁻^1^; −5.54% for 125 kg·m⁻^2^·s⁻^1^). Mechanistically, weak pitching accelerates flow boiling via inertial force augmentation, while excessive pitching disrupts phase distribution, inducing deterioration. Optimal heat transfer occurred at 2.5° amplitude, 0.2 Hz frequency, and 175 kg·m⁻^2^·s⁻^1^, with peak enhancements consistently observed in low-to-medium frequency ranges (0.2–0.4 Hz) across amplitudes. At low frequencies (0.2–0.4 Hz), periodic inertial forces generate flow perturbations that enhance turbulent mixing, and promote bubble detachment—mechanisms amplified at moderate mass fluxes (175 kg·m⁻^2^·s⁻^1^) where shear stress synergizes with inertial forces to intensify nucleate boiling. The 175 kg·m⁻^2^·s⁻^1^ optimal condition reflects balanced inertial perturbation and mass flux-driven shear, maximizing interfacial heat transfer without inducing flow instability. Conversely, lower mass flux (125 kg·m⁻^2^·s⁻^1^) requires higher frequency (0.4 Hz) to generate sufficient perturbation, while higher flux (225 kg·m⁻^2^·s⁻^1^) is less sensitive to inertial forces due to dominant shear-driven turbulence.

#### Influence of pitching acceleration on heat transfer performance

**Fig. 8:**
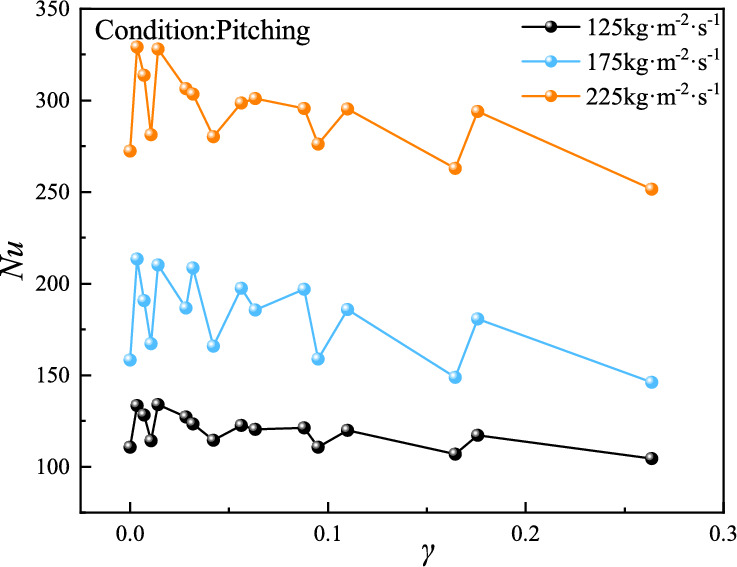
The variation of heat transfer performance with sloshing intensity under pitching motion.

The experimental results of convective heat transfer in the plate evaporator under simulated UUV pitching motion (Fig. 7) reveal that both pitching frequency and amplitude synergistically affect the convective heat transfer coefficient, which is characterized using the dimensionless sloshing intensity γ and Nusselt number Nu (Fig. 8). For mass flux of 175 and 225 kg·m⁻^2^·s⁻^1^, sloshing intensity dominates heat transfer behavior: Nu exhibits oscillatory increases in the low γ range (0–0.1) with peak enhancement at γ = 0.003 (34.75% and 20.77%, respectively), followed by oscillatory decreases in medium-to-high γ (0.1–0.26) and maximum attenuation at γ = 0.26 (−7.78% for both). In contrast, at 125 kg·m⁻^2^·s⁻^1^, Nu oscillates within low γ (0–0.1) but stabilizes in medium-to-high γ (0.1–0.26), where mass flux becomes the dominant factor. Mechanistically, weak pitching accelerates flow boiling via augmented inertial forces, enhancing heat transfer, while strong pitching disrupts gas–liquid distribution by limiting axial inertial forces, inducing deterioration. Optimal heat transfer occurs at γ = 0.003 and 175 kg·m⁻^2^·s⁻^1^, whereas the most unfavorable condition is observed at γ = 0.26 and 175 kg·m⁻^2^·s⁻^1^. The observed trends stem from γ-dependent modulation of inertial forces governing two-phase flow dynamics. The higher mass fluxes (225 kg·m⁻^2^·s⁻^1^) maintain turbulent mixing despite inertial instability. The optimal γ = 0.003 represents a critical balance between inertial perturbation intensity and flow stability.

#### Influence of pitching motion on the total heat transfer coefficient

**Fig. 9:**
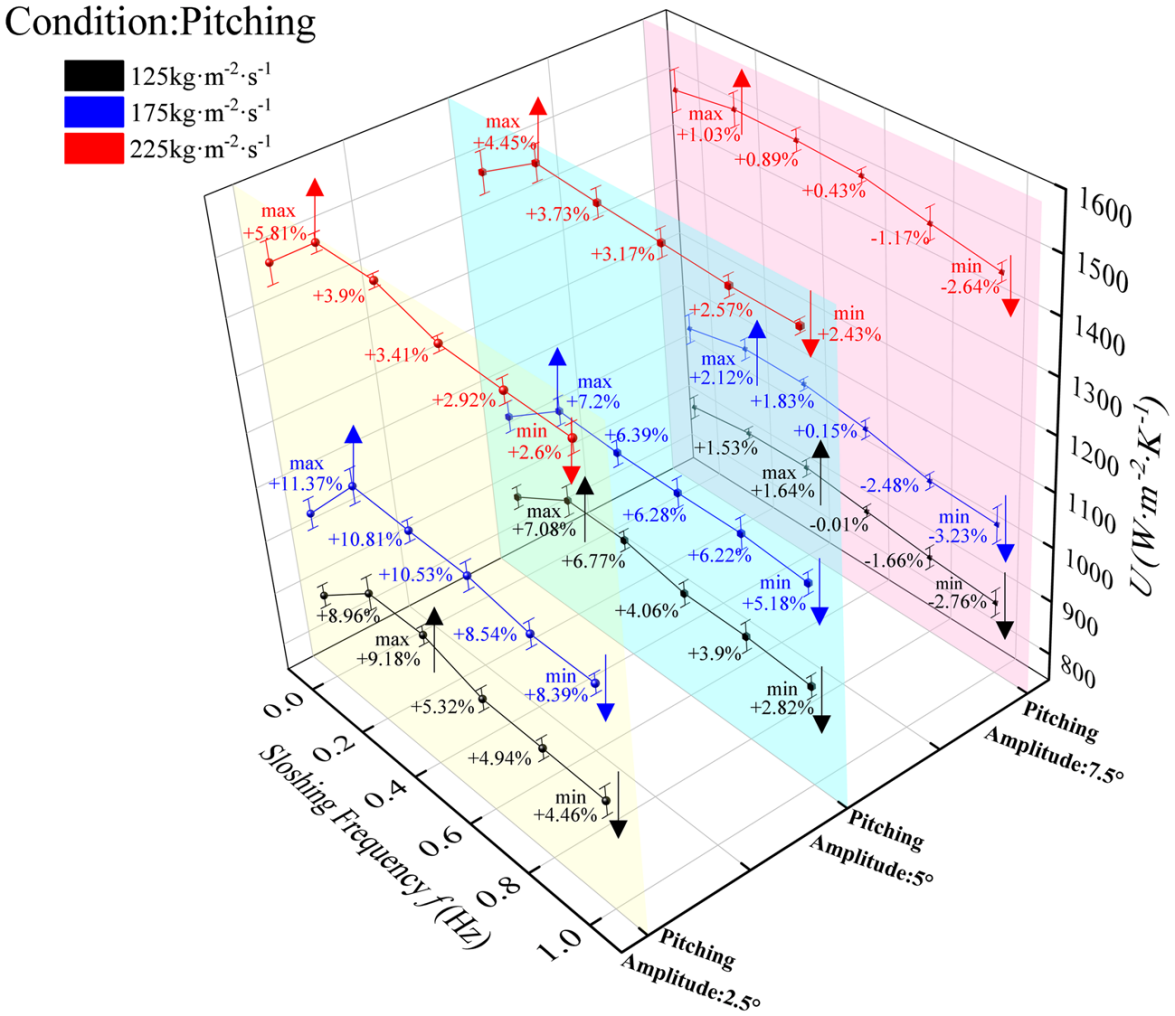
Total heat transfer coefficient of plate heat exchanger under pitching motion.

The total heat transfer coefficient (HTC) of the plate evaporator under varying pitching conditions (Fig. 9) exhibits frequency-dependent behavior consistent with the convective heat transfer coefficient trends in Fig. 7. At 1 Hz, minimum HTC values were observed across all amplitudes, though performance remained superior to stable conditions (0 Hz) for 2.5° and 5° amplitudes: at 2.5°, enhancements were 4.46% (125 kg·m⁻^2^·s⁻^1^), 8.39% (175 kg·m⁻^2^·s⁻^1^), and 2.6% (225 kg·m⁻^2^·s⁻^1^); at 5°, marginal improvements persisted (2.82%, 5.18%, 2.43% for the same flux). Conversely, 7.5° amplitude induced heat transfer deterioration at 1 Hz (−2.76%, −3.23%, −2.64% vs. stable conditions). For 2.5° and 5° amplitudes, HTC response followed a unified pattern: significant enhancement under smooth (0–0.2 Hz) and harsh (0.2–0.5 Hz) motion, with attenuation in extreme frequencies (0.6–1 Hz) where mass flux dominated; notably, 2.5° amplitude yielded stronger enhancements than 5°. At 7.5°, overall HTC reduction occurred, with sloshing conditions becoming the dominant factor governing heat transfer performance. At low-to-moderate amplitudes (2.5°–5°), periodic inertial forces generate controlled perturbations that enhance turbulent mixing, and promote bubble detachment—effects amplified by synergistic coupling between shear stress and inertial perturbations at 175 kg·m⁻^2^·s⁻^1^, explaining the peak enhancement. The frequency-dependent transition (enhancement at 0–0.5 Hz vs. weakening at 0.6–1 Hz) reflects a critical balance. Mass flux dominance at extreme frequencies arises from insufficient shear stress to sustain perturbation-driven mixing, whereas sloshing intensity dominates at 7.5° amplitude due to overwhelming inertial disruption of phase distribution.

### Influence of rolling motion

#### Influence of rolling motion on convective heat transfer performance

**Fig. 10:**
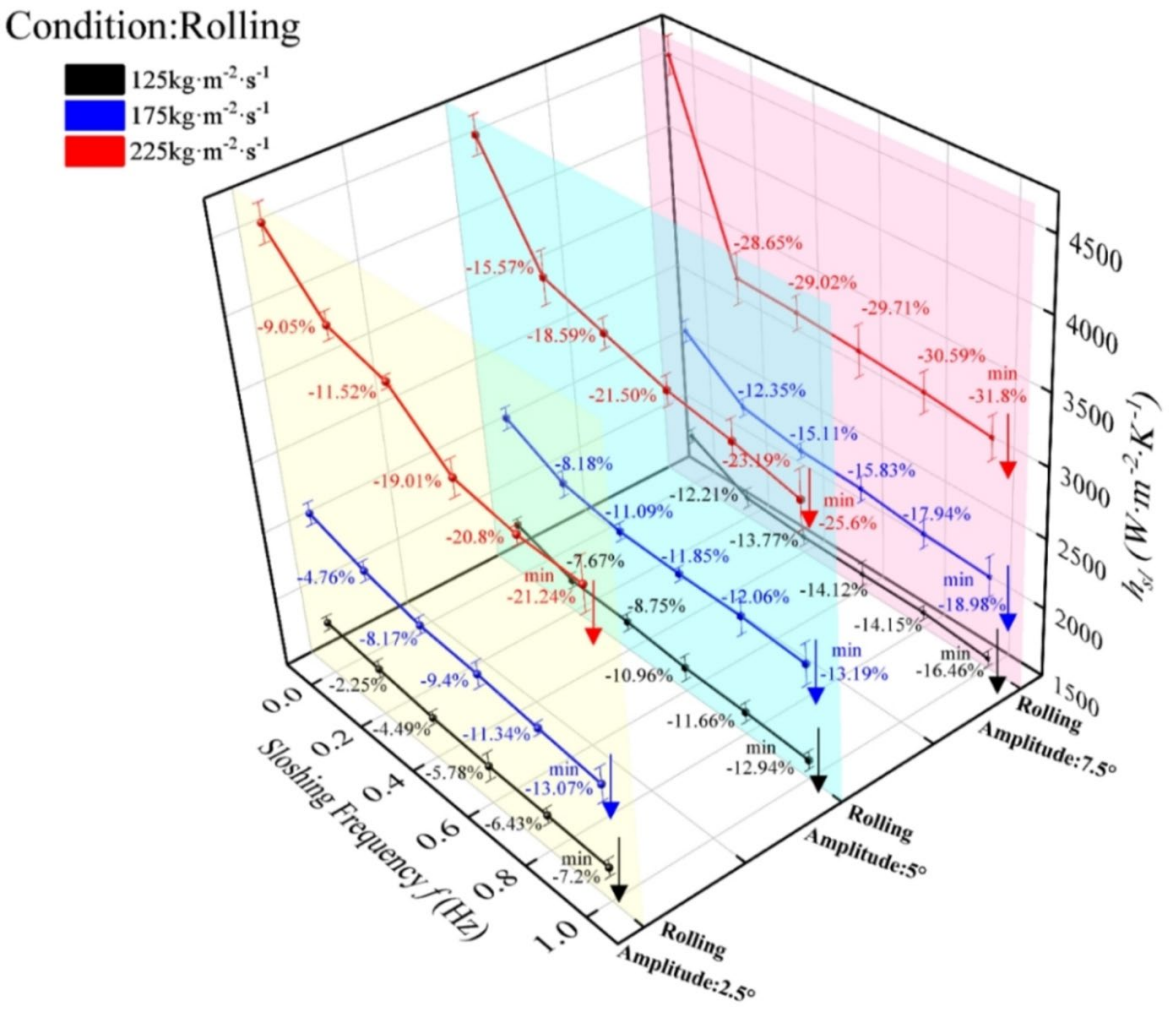
Convective heat transfer coefficient of R134a under rolling motion.

The experimental results of convective heat transfer in the plate evaporator under UUV rolling motion (Fig. 10) demonstrate consistent trends across mass flux: the convective heat transfer coefficient (HTC) decreases monotonically with rolling frequency, reaching minimum values for R134a at 1 Hz. Under constant mass flux, HTC attenuation intensifies with increasing rolling amplitude, while at fixed amplitude, higher mass flux exacerbate heat transfer weakening. Specifically, minimum HTC values at 1 Hz exhibited amplitude-dependent reductions: 2.5° (7.2%, 13.07%, 21.24% for 125/175/225 kg·m⁻^2^·s⁻^1^), 5° (12.94%, 13.19%, 25.6%), and 7.5° (16.46%, 18.98%, 31.8%). Mechanistically, rolling-induced inertial forces likely delay the working fluid’s flow boiling process, consistently weakening heat transfer with increasing amplitude and frequency. The most severe deterioration occurred at 7.5° amplitude, 1 Hz frequency, and 225 kg·m⁻^2^·s⁻^1^ mass flux. The amplitude-dependent exacerbation reflects intensified inertial instability with larger displacements, while mass flux-dependent degradation (most severe at 225 kg·m⁻^2^·s⁻^1^) arises from synergistic coupling between high shear stress and inertial perturbations, which amplifies flow maldistribution rather than enhancing mixing. The monotonic frequency effect indicates a lack of the critical balance between perturbation intensity and flow stability observed in pitching conditions, resulting in unmitigated heat transfer impairment across the tested parameter range.

#### Influence of rolling acceleration on heat transfer performance

**Fig. 11:**
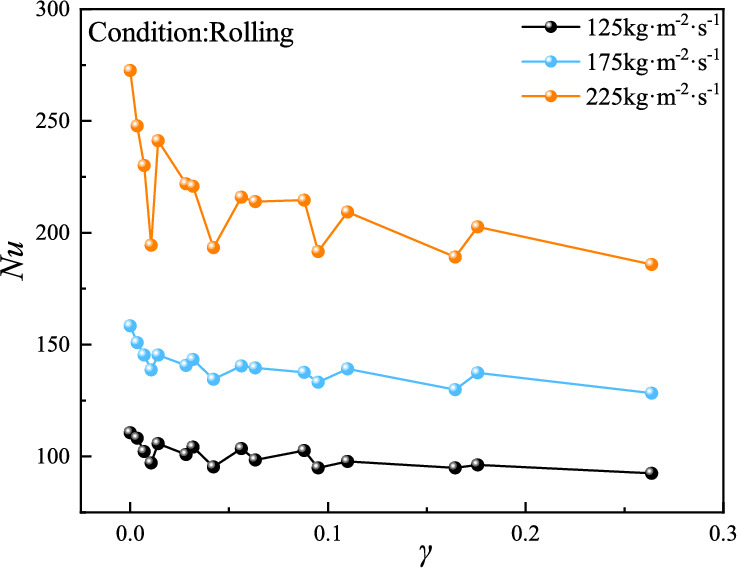
The variation of heat transfer performance with sloshing intensity under rolling motion.

The experimental results of convective heat transfer in the plate evaporator under UUV rolling motion (Fig. 10) demonstrate that both rolling frequency and amplitude synergistically affect the convective heat transfer coefficient, characterized using the dimensionless sloshing intensity γ and Nusselt number Nu (Fig. 11). Compared to stable conditions (γ = 0), rolling consistently weakens heat transfer performance across mass flux: Nu decreases linearly with γ in the 0–0.014 range, followed by oscillatory decay between 0.028–0.26, with maximum deterioration at γ = 0.26 (16.46%, 18.98%, and 31.8% reductions for 125, 175, and 225 kg·m⁻^2^·s⁻^1^, respectively). Notably, the Nu-sloshing intensity relationship is most pronounced at 225 kg·m⁻^2^·s⁻^1^ compared to lower flux. Sloshing intensity emerges as the dominant factor, as rolling-induced inertial forces oppose the working fluid’s axial momentum, causing flow lag and restricting flow boiling. The most severe heat transfer deterioration occurs at γ = 0.26 and 225 kg·m⁻^2^·s⁻^1^. The linear Nu decline at low γ (0–0.014) reflects initial flow destabilization, while oscillatory behavior at higher γ (0.028–0.26) indicates intermittent flow regime transitions. The pronounced effect at 225 kg·m⁻^2^·s⁻^1^ arises from synergistic coupling between high shear stress and inertial perturbations, exacerbating flow maldistribution rather than promoting turbulence. The dominance of sloshing intensity confirms that inertial disruption of phase distribution—rather than mass flux or frequency-dependent stability balance—governs heat transfer performance under rolling conditions.

#### Influence of rolling motion on the total heat transfer coefficient

**Fig. 12:**
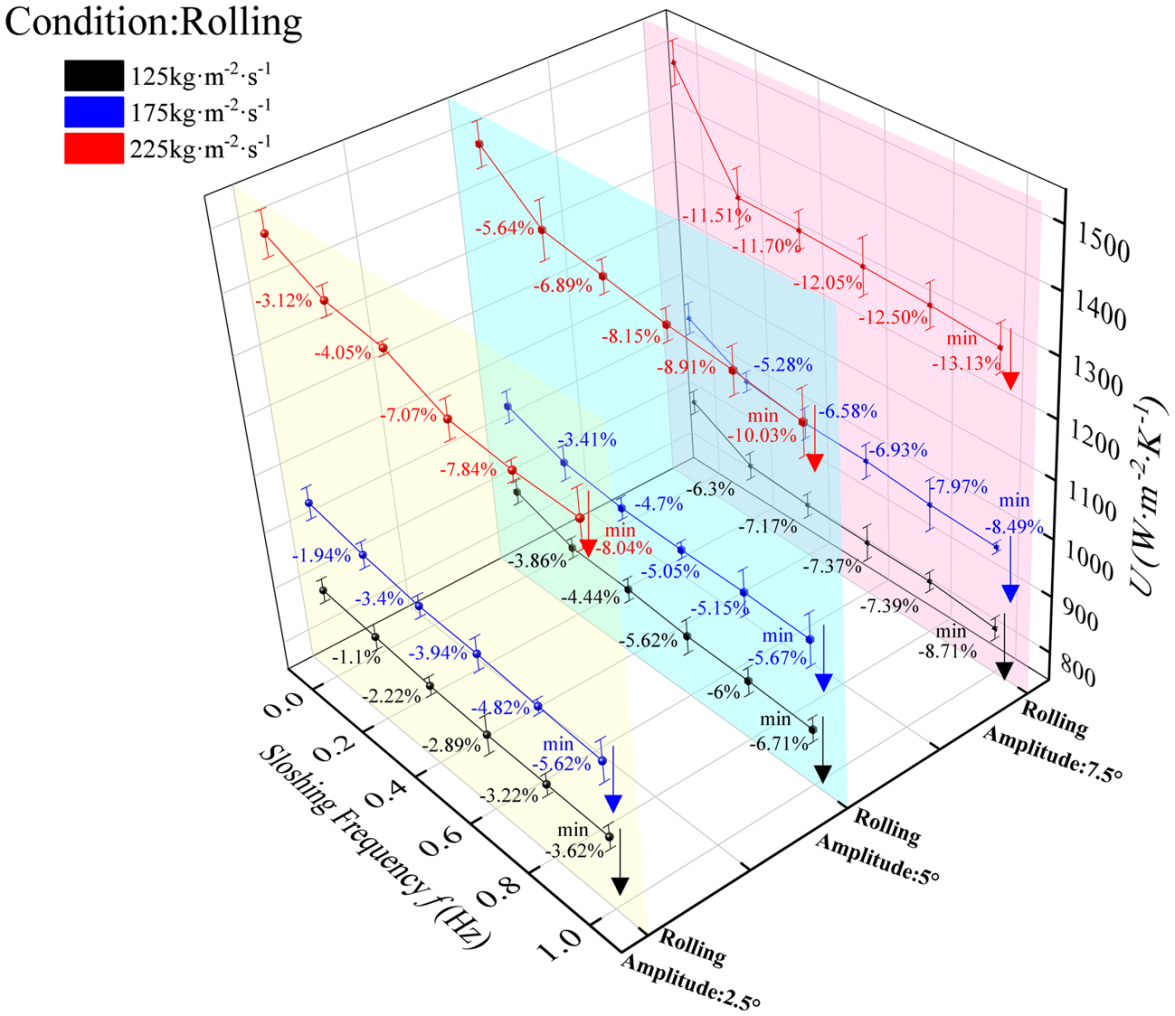
Total heat transfer coefficient of plate heat exchanger under rolling motion.

The total heat transfer coefficient (HTC) of the plate evaporator under rolling conditions (Fig. 12) exhibits frequency-dependent decay consistent with convective heat transfer trends in Fig. 10, with minimum values at 1 Hz across all amplitudes—all inferior to stable (0 Hz) performance. At 2.5° amplitude, HTC reductions were 3.62% (125 kg·m⁻^2^·s⁻^1^), 5.62% (175 kg·m⁻^2^·s⁻^1^), and 8.04% (225 kg·m⁻^2^·s⁻^1^); at 5°, deteriorations intensified to 6.71%, 5.67%, and 10.03% for the same flux; maximal attenuation occurred at 7.5° (−8.71%, −8.49%, −13.13%). Across amplitudes, HTC decreased monotonically with rolling frequency, with motion conditions emerging as the dominant factor governing heat transfer deterioration. Unlike convective heat transfer coefficient, the total heat transfer coefficient integrates conductive and convective resistances; however, the observed monotonic decline with rolling frequency indicates convective suppression as the primary driver, with negligible contribution from conductive resistance changes (e.g., wall conduction remains unaffected by inertial forces). The amplitude-dependent exacerbation (most severe at 7.5°) reflects intensified inertial instability, while mass flux-dependent degradation (maximum at 225 kg·m⁻^2^·s⁻^1^) arises from synergistic coupling between high shear stress and inertial perturbations, which amplifies flow maldistribution rather than enhancing turbulence. The dominance of motion conditions confirms that inertial disruption governs the overall heat transfer performance under rolling motion.

## Development of flow boiling heat transfer correlation for plate heat exchanger under different motion

Previous studies demonstrate that the heaving, pitching, and rolling motions of UUVs (Unmanned Underwater Vehicles) significantly impact the heat transfer performance of the plate evaporator in their ocean thermal energy conversion systems. To accurately describe the influence of different motion conditions on heat transfer performance, Chen and Hu respectively proposed a calculation method^35,38^, namely Eqs. (6)-(7). This set of equations indicates that the correction model is divided into two parts: one part is the convective heat transfer model under stable conditions, and the other part is the correction factor *Fsl*​ under sloshing conditions.

6 $${h}_{sl}={F}_{sl}{h}_{st}$$

7 $${h}_{sl}={F}_{sl}{h}_{st}={h}_{st}\left({C}_{1}\left({G}_{r}/{G}_{r}{\prime}\right)\cdot {C}_{2}\left({A}_{sl}/{A}_{sl}{\prime},{t}_{0}/{t}_{0}{\prime}\right)+1\right)$$

This study introduces the concept of sloshing intensity to characterize the impact of heaving, pitching, and rolling motions on heat transfer performance. The introduction of sloshing intensity replaces the use of motion amplitude and frequency to jointly represent the impact of motion on heat transfer performance as described in the aforementioned calculation methods^35,38^. Equation (10) has been revised to Eqs. (8) and (9).

8 $${C}_{1}\left({G}_{r}/{G}_{r}{\prime}\right)={a}_{1}\cdot {\left({G}_{r}/{G}_{r}{\prime}\right)}^{b}+{a}_{2}$$

9 $${C}_{2}\left({a}_{sl}/{a}_{sl}{\prime}\right)={e}^{{a}_{3}\left(\gamma +1\right)}$$

The two-phase convective heat transfer coefficient under stable conditions can be calculated using existing heat transfer correlations for plate evaporators. In this paper, the Yan and Lin model^39^ is adopted as a semi-empirical model and extended to our experiment, as shown in Eqs. (10)-(11):

10 $${h}_{st}=\frac{Nu{\lambda }_{r}}{{d}_{e}}$$

11 $${\mathrm{Nu}}={\alpha }_{1}{Pr}^\frac{1}{3}{\mathrm{Bi}}_{r}^{{\alpha }_{2}}{\mathrm{Re}}_{r}^{{\alpha }_{3}}[(1-x)+x(\frac{{p}_{liq}}{{p}_{gas}}{)}^{0.5}{]}^{{\alpha }_{4}}$$

In the equations, *h*_*st*_ is the convective heat transfer coefficient of the plate evaporator under stable conditions, which includes the Nusselt number *Nu*, the thermal conductivity of the working fluid *λ*_*r*_, and the equivalent diameter of the plate evaporator *d*_*e*_. The coefficients *α*_*1*_, *α*_2_, *α*_3_, and *α*_4_ in Eq. (14) are obtained by fitting experimental data. Table 4 shows the fitting results.

**Table 4:** Fitting of correlation coefficients under stable conditions.

Working fluid	*α*_1_	*α*_2_	*α*_3_	*α*_4_
R134a	1.24	0.697	0.5	0.47

The sloshing correction factor *F*_*sl*_ is the key aspect of the heat transfer correlation proposed in this study.*F*_*sl*_ is a function of mass flux *G*_*r*_ and motion intensity *γ*. *F*_*sl*_ consists of two parts, *C*_*1*_ and *C*_*2*_. *C*_*1*_ reflects the flow regime transition of different mass flux fluids under sloshing conditions and is a function of mass flux *G*_*r*_, with *G*_*r*_′ as the reference value, taken as 125 kg·m^−2^·s^−1^ in this study. *C*_*2*_ reflects the impact of sloshing conditions on heat transfer performance, with the parameter being sloshing intensity *γ*. The reference value *g* is taken as 9.8 m·s^−2^ in this study. *H*_*sl*_ reflects the flow regime transition is the convective heat transfer coefficient of R134a under different heaving, pitching, and rolling conditions. The coefficients *a*_*1*_, *a*_*2*_, *a*_*3*_ and *b* can be obtained by fitting experimental data. These coefficients *a*_*1*_, *a*_*2*_, *a*_*3*_ and *b* are derived from nonlinear fitting based on the experimental results of this study, encompassing heaving, pitching, and rolling motion conditions. The fitting results are shown in Table 5.

**Table 5:** Fitting of correlation coefficients under sloshing.

Working fluid	Condition	*a*_*1*_	*a*_*2*_	*a*_*3*_	*b*
R134a	heaving	0.07	0.12	0.4	1
	pitching	0.44	−0.16	−0.5	−0.6
	rolling	0.22	−0.65	−0.83	−0.67

**Fig. 13:**
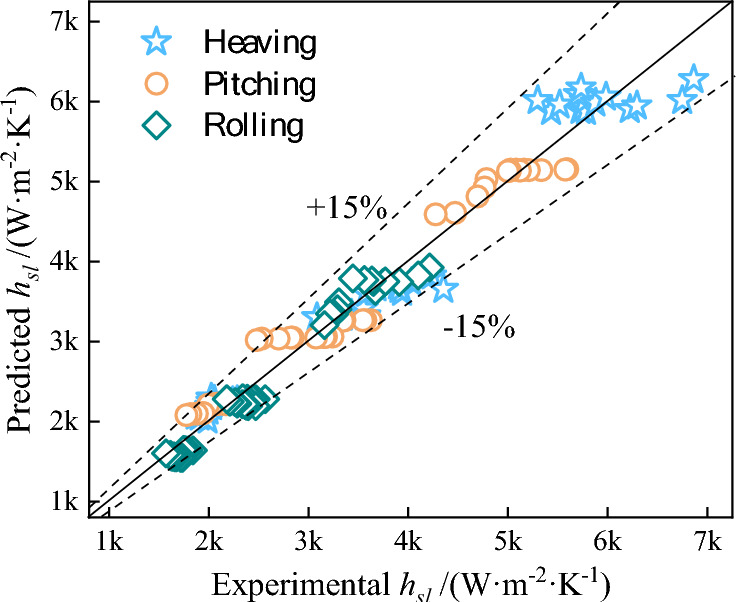
Comparison of experimental data with predicted values.

Figure 13 compares the calculated values of the heat transfer correlation for flow boiling in the plate evaporator under heaving, pitching, and rolling conditions with the experimental data. The fitted heat transfer correlation predictions agree well with the experimental values, with an error within ± 15%. It should be noted that the inlet working fluid of the plate evaporator in this experiment is saturated, and the vapor quality of the outlet working fluid is 0.3–0.4. And also, it should be noted that the applicable range of the heat transfer correlation established in this study is for pitching and rolling amplitudes of 2.5° to 7.5°, heaving amplitudes of 50 to 100 mm, frequencies of 0 to 1 Hz, mass fluxes of 125 to 225 kg·m^−2^·s^−1^, and the working fluid being R134a.

## Conclusions

To investigate variations in the heat transfer performance of R134a plate evaporators within Unmanned Underwater Vehicles (UUVs) ocean thermal energy conversion systems under marine motions, this study employed a 6-DOF motion platform to simulate heaving, pitching, and rolling conditions, yielding the following key findings: Heaving motion significantly enhanced R134a convective heat transfer, with a maximum increase of 61.8% observed at an amplitude of 100 mm and frequency of 0.6 Hz; heat transfer coefficients initially increased with sloshing frequency, peaked at 0.6 Hz, and subsequently decreased. For heaving motion, synchronized vertical acceleration amplifies flow perturbations aligned with the working fluid direction, enhancing turbulence intensity—key mechanisms for convective heat transfer augmentation. The frequency-dependent peak (0.6 Hz) reflects resonance between motion frequency and flow oscillation, beyond which excessive inertial forces cause flow separation and reduce heat transfer efficiency.

For pitching motion, low-amplitude (2.5°) conditions improved performance (maximum 34.75% enhancement at 0.2 Hz), whereas high-amplitude (7.5°) motion reduced it (maximum 7.79% decrease at 1 Hz), with a similar frequency-dependent peak at 0.2 Hz, indicating optimal amplitude-frequency combinations for both heaving and pitching that balance disturbance intensity and flow stability to maximize heat transfer enhancement. Pitching motion exhibits amplitude-dependent duality: low amplitudes introduce mild secondary flows that promote mixing without disrupting phase distribution, while high amplitudes generate stratified flow and localized dry-out regions that degrade performance.

In contrast, rolling motion strongly degraded performance, with a maximum 31.8% decrease at 7.5° amplitude and 1 Hz frequency, where coefficients decreased monotonically with increasing frequency. Rolling motion, by imposing lateral inertial forces opposing the primary flow direction, induces periodic flow reversal and phase maldistribution, suppressing bubble nucleation and detachment—critical processes for boiling heat transfer—thus causing monotonic performance degradation with frequency. The sloshing intensity parameter (γ) quantifies these inertial effects, linking motion kinematics to flow stability and heat transfer efficiency through its influence on turbulence generation and thermal boundary layer characteristics. Finally, a heat transfer correlation incorporating a sloshing correction factor (Fsl) was developed, demonstrating good agreement with experimental data (± 15% deviation), thus providing critical insights for optimizing UUV thermal system design under marine motion conditions. It should be noted that the applicable range of the heat transfer correlation established in this study is for pitching and rolling amplitudes of 2.5° to 7.5°, heaving amplitudes of 50 to 100 mm, frequencies of 0 to 1 Hz, mass fluxes of 125 to 225 kg·m-2·s-1, and the working fluid being R134a.

## Supplementary Information

Supplementary Information.

## Data Availability

The datasets used and/or analysed during the current study available from the correspondingauthor on reasonable request.

## Abbreviations

*A*: Heat transfer area(m^2^)
*AMP*: Amplitude
*A*_*sl*_: Amplitude value
$A_{sl}{'}$: Reference Amplitude value
*a*_*sl*_: Sloshing acceleration(m·s^-2^)
$a_{sl}{'}$: Reference Sloshing acceleration(m·s^-2^)
*Bi*: Biot number
*c*_*p*__*,*__*w*__*ater*_: The specific heat capacity of hot water at constant pressure (J·kg^-1^·K^-1^)
*ρ*_*liq*_: Liquid phase working fluid density(kg·m^-3^)
*d*_*e*_: Equivalent diameter(m)
*F*_*sl*_: Sloshing correction factor
*G*_*w*__*ater*_: Mass flux of water(kg·m^-2^·s^-1^)-
*G*_*r*_: Mass flux of refrigerant (kg·m^-2^·s^-1^)
$G{'}$: Reference Mass flux(kg·m^-2^·s^-1^)
*g*: Acceleration of gravity(m·s^-2^)
*h*_*w*__*ater*__*,sat*_: Side heat transfer coefficient of water (W·m^-2^·K^-1^)
*h*_*r,sat*_: Saturated evaporation convection heat transfer coefficient(W·m^-2^·K^-1^)
*h*_*sl*_: Convective heat transfer coefficient under sloshing condition(W·m^-2^·K^-1^)
*h*_*st*_: Convective heat transfer coefficient at steady state(W·m^-2^·K^-1^)
*Nu*: Nusselt number
*Pr*: Prandtl number
*Re*: Reynolds number
LMTD(*ΔT*_*m*_): Log-mean temperature difference between fluids(K)-
*t*_*0*_: Sloshing period
$$t_0{'}$$: Reference period
*T*_*e*_: Evaporation temperature(K)
*T*_*hot*__*,*__*water,*__*in*_: Inlet temperature of hot water(K)
*T*_*hot*__*,*__*water,*__*out*_: Outlet temperature of hot water(K)
*U*: Total heat transfer coefficient of the heat exchanger(W·m^-2^·K^-1^)
*U*_*sat*_: Total heat transfer coefficient at saturation evaporation(W·m^-2^·K^-1^)
*x*: Vapor quality
*ρ*_*gas*_: Gass phase working fluid density(kg·m^-3^)

## Greek symbols

*α*: Coefficient
*λ*: Thermal conductivity(W·m^-1^·K^-1^)
*δ*: Thickness(m)
*ρ*: Density(kg·m^-3^)
*γ*: Sloshing intensity

## Subscripts

*pl*: Plate
*sl*: Under sloshing conditions
*r*: Refrigerant
*sat*: Saturation state
*in*: Inlet
*out*: Outlet
*st*: Stability condition

## References

[1] Li, X. et al. Modeling and system analysis of floating underwater vehicle with variable mass and center of gravity. *Ocean Eng.* **267**, 113303. 10.1016/j.oceaneng.2022.113303 (2023).

[2] Rigaud, V. Innovation and Operation with robotized systems. *IFAC Proc. Vol.* **42**(18), 44–51. 10.3182/20090916-3-BR-3001.0076 (2009).

[3] Ji, D. H. et al. Design and control of hybrid underwater glider. *Adv. Mech. Eng.* **11**(5), 1687814019848556. 10.1177/1687814019848556 (2019).

[4] Chen, Y. et al. Efficiency and power density analysis on phase change material-based ocean thermoelectric generator for underwater vehicle. *J. Energy Storage* **89**, 111686. 10.1016/j.est.2024.111686 (2024).

[5] Ellabban, O., Abu-Rub, H. & Blaabjerg, F. Renewable energy resources: Current status, future prospects and their enabling technology. *Renew. Sustain. Energy Rev.* **39**, 748–764. 10.1016/j.rser.2014.07.113 (2014).

[6] Martínez, M. L. et al. A systemic view of potential environmental impacts of ocean energy production. *Renew. Sustain. Energy Rev.* **149**, 111332. 10.1016/j.rser.2021.111332 (2021).

[7] Wang, X. et al. Reviews of power systems and environmental energy conversion for unmanned underwater vehicles. *Renew. Sustain. Energy Rev.* **16**(4), 1958–1970. 10.1016/j.rser.2011.12.016 (2012).

[8] Wang, G., Yang, Y. & Wang, S. Ocean thermal energy application technologies for unmanned underwater vehicles: A comprehensive review. *Appl. Energy* **278**, 115752. 10.1016/j.apenergy.2020.115752 (2020).

[9] Hu, Z., Chen, Y. & Zhang, C. Role of R717 blends in ocean thermal energy conversion organic Rankine cycle. *Renew. Energy* **221**, 119756. 10.1016/j.renene.2023.119756 (2024).

[10] Fan, C., Zhang, C. & Gao, W. Improving the ocean thermal energy conversion by solar pond. *Sol. Energy* **274**, 112583. 10.1016/j.solener.2024.112583 (2024).

[11] Hu, Z. & Chen, Y. Advancements in sustainable desalination with ocean thermal energy: A review. *Desalination* **586**, 117770. 10.1016/j.desal.2024.117770 (2024).

[12] Fan, C., Wu, Z., Wang, J., Chen, Y. & Zhang, C. Thermodynamic process control of ocean thermal energy conversion. *Renew. Energy* **210**, 810–821. 10.1016/j.renene.2023.04.029 (2023).

[13] Hu, Z., Deng, Z., Gao, W. & Chen, Y. Experimental study of the absorption refrigeration using ocean thermal energy and its under-lying prospects. *Renew. Energy* **213**, 47–62. 10.1016/j.renene.2023.05.086 (2023).

[14] Zhang, J., Zhu, X., Mondejar, M. E. & Haglind, F. A review of heat transfer enhancement techniques in plate heat exchangers. *Renew. Sustain. Energy Rev.* **101**, 305–328. 10.1016/j.rser.2018.11.017 (2019).

[15] Isa, K., Arshad, M. R. & Ishak, S. A hybrid-driven underwater glider model, hydrodynamics estimation, and an analysis of the motion control. *Ocean Eng.* **81**, 111–129. 10.1016/j.oceaneng.2014.02.002 (2014).

[16] Nilpueng, K. & Wongwises, S. Two-phase gas–liquid flow characteristics inside a plate heat exchanger. *Exp. Thermal Fluid Sci.* **34**(8), 1217–1229. 10.1016/j.expthermflusci.2010.05.001 (2010).

[17] Wang, Z. et al. Bionic microchannels for step lifting transpiration. *Int. J. Extreme Manuf.* **5**(2), 025502. 10.1088/2631-7990/acbcff (2023).

[18] Trimulyono, A., Hashimoto, H. & Matsuda, A. Experimental validation of single- and two-phase smoothed particle hydrodynamics on sloshing in a prismatic tank. *J. Marine Sci. Eng.* **7**(8), 247. 10.3390/jmse7080247 (2019).

[19] Zheng, W., Cai, W. & Jiang, Y. Distribution characteristics of gas-liquid mixture in spiral-wound heat exchanger under sloshing conditions. *Cryogenics* **99**, 68–77. 10.1016/j.cryogenics.2019.03.001 (2019).

[20] Yuan, H., Tan, S., Zhuang, N. & Lan, S. Flow and heat transfer in laminar–turbulent transitional flow regime under rolling motion. *Ann. Nucl. Energy* **87**, 527–536. 10.1016/j.anucene.2015.10.009 (2016).

[21] Walraven, D., Laenen, B. & D’haeseleer, W. Comparison of shell-and-tube with plate heat exchangers for the use in low-temperature organic Rankine cycles. *Energy Convers. Manag.* **87**, 227–237. 10.1016/j.enconman.2014.07.019 (2014).

[22] Huang, J., Sheer, T. J. & Bailey-McEwan, M. Heat transfer and pressure drop in plate heat exchanger refrigerant evaporators. *Int. J. Refrig* **35**(2), 325–335. 10.1016/j.ijrefrig.2011.11.002 (2012).

[23] Khan, M. S., Khan, T. S., Chyu, M.-C. & Ayub, Z. H. Evaporation heat transfer and pressure drop of ammonia in a mixed configuration chevron plate heat exchanger. *Int. J. Refrig* **41**, 92–102. 10.1016/j.ijrefrig.2013.12.015 (2014).

[24] Lee, H., Hwang, Y., Radermacher, R. & Chun, H.-H. Thermal and hydraulic performance of sinusoidal corrugated plate heat exchanger for low temperature lift heat pump. *Int. J. Refrig.* **36**(3), 689–700. 10.1016/j.ijrefrig.2012.11.008 (2013).

[25] Han, D., Lee, K. & Kim, Y. Experiments on the characteristics of evaporation of R410A in brazed plate heat exchangers with different geometric configurations. *Appl. Therm. Eng.* **23**(10), 1209–1225. 10.1016/S1359-4311(03)00061-9 (2003).

[26] Chen, Y. P. & Deng, Z. L. Hydrodynamics of droplet passing through a microfluidic T-junction. *J. Fluid Mech.* **819**, 401–434 (2017).

[27] Chen, Y. P., Liu, X. D. & Shi, M. H. Hydrodynamics of double emulsion droplet in shear flow. *Appl. Phys. Lett.* **102**(5), 051609 (2013).

[28] Li, W. M., Yang, S. Y., Chen, Y. P., Li, C. & Wang, Z. K. Tesla valves and capillary structures-activated thermal regulator. *Nat. Commun.* **14**, 3996 (2023). 37414775 10.1038/s41467-023-39289-5 PMC10325955

[29] Liu, X. D., Hao, G. Q., Li, B. & Chen, Y. P. Experimental study on the electrohydrodynamic deformation of droplets in a combined DC electric field and shear flow field. *Fundam. Res.* **3**(2), 274–287 (2023). 38932921 10.1016/j.fmre.2021.10.011 PMC11197815

[30] Dong, L., Dong, C. & Wu, X. Numerical simulation of heat transfer performance of spiral wound heat exchanger under sloshing condition. *PLoS ONE* **18**(12), e0295315. 10.1371/journal.pone.0295315 (2023). 38079437 10.1371/journal.pone.0295315 PMC10712881

[31] Zheng, W. et al. Sloshing effect on gas-liquid distribution performance at entrance of a plate-fin heat exchanger. *Exp. Thermal Fluid Sci.* **93**, 419–430. 10.1016/j.expthermflusci.2018.01.012 (2018).

[32] Du, X. et al. Research on shell-side heat and mass transfer with multi-component in LNG spiral-wound heat exchanger under sloshing conditions. *Pet. Sci.* **21**(2), 1333–1345. 10.1016/j.petsci.2023.10.016 (2024).

[33] Ren, Y., Cai, W. & Jiang, Y. Numerical study on shell-side flow and heat transfer of spiral-wound heat exchanger under sloshing working conditions. *Appl. Therm. Eng.* **134**, 287–297. 10.1016/j.ijheatmasstransfer.2018.01.025 (2018).

[34] Hu, H., Li, J., Li, Y. & Xie, Y. Experimental investigation on flow boiling characteristics in offset strip fin channels under different sloshing conditions. *Exp. Thermal Fluid Sci.* **145**, 110880. 10.1016/j.expthermflusci.2023.110880 (2023).

[35] Hu, H., Li, Y. & Li, J. Experimental investigation on flow boiling characteristics in zigzag channels under different sloshing conditions. *Appl. Therm. Eng.* **230**, 120780. 10.1016/j.applthermaleng.2023.120780 (2023).

[36] Zhou, F., Lu, T., Zhuang, D. & Ding, G. Experimental study of condensation heat transfer characteristics on metal foam wrapped tubes under sloshing conditions. *Int. J. Heat Mass Transf.* **176**, 121394. 10.1016/j.ijheatmasstransfer.2021.121394 (2021).

[37] Fei, S., Jiang, H. & Hua, D. Study on boiling heat transfer characteristics of R410A outside horizontal tube under swaying condition. *Sci. Rep.* **14**(1), 1979. 10.1038/s41598-024-52568-5 (2024). 38263440 10.1038/s41598-024-52568-5 PMC11294476

[38] Chen, C. et al. Heat transfer characteristics of oscillating flow in a narrow channel under rolling motion. *Ann. Nucl. Energy* **110**, 668–678. 10.1016/j.anucene.2017.07.019 (2017).

[39] Hsieh, Y. Y. & Lin, T. F. Evaporation heat transfer and pressure drop of refrigerant R410A flow in a vertical plate heat exchanger. *J. Heat Transfer* **125**(5), 852–857. 10.1115/1.1518498 (2003).

