# Supplementary material for: Experimental study on heat transfer characteristics of plate evaporator under heaving, pitching, and rolling conditions
Source: Sci Rep. 2025 Dec 10;16:1998. doi: 10.1038/s41598-025-31723-6 (PMC12808697; doi:10.1038/s41598-025-31723-6)
Supplement: Supplementary file 1 — Supplementary Information. [file 41598_2025_31723_MOESM1_ESM.docx]

**Appendix A**

When a plate heat exchanger is used as an evaporator or condenser in an ocean thermal energy conversion system, seawater, acting as the heat source or cold source, flows in a single phase within the channels on one side of the plate heat exchanger and exchanges heat with the phase-change working fluid in the channels on the other side. Since there are differences in the corrugated structure and geometric dimensions of the heat exchange plates in different plate heat exchangers, the general single-phase heat transfer correlation cannot meet the accurate calculation of the single-phase convective heat transfer coefficient of the plate heat exchanger in this study. Therefore, based on the improved Wilson plot method, this paper fits and obtains a single-phase heat transfer correlation suitable for the plate heat exchanger in this study.

To improve the accuracy of solving heat transfer correlations, various correction methods have been developed on the basis of the traditional Wilson plot method. Many scholars have fitted and solved the single-phase heat transfer correlations of heat exchangers based on heat transfer models where the heat transfer coefficient on one side is a function and that on the other side is a constant, models where both hot and cold side heat transfer coefficients are functions, and models with multiple undetermined coefficients .Among them, the heat transfer model where both the cold and hot side heat transfer coefficients are in the form of functions describes the heat transfer process more accurately and is easier to solve. Therefore, this paper uses this correction method to fit and solve the single-phase heat transfer correlation of the plate heat exchanger. The heat transfer model and specific solution process are as follows:

The total thermal resistance *R_tot_* of the plate heat exchanger consists of the convective thermal resistance of the cold fluid *R_cold_*, the convective thermal resistance of the hot fluid *R_hot_*, and the thermal conduction resistance of the plate *R_wall_*:

$$R_{tot}=R_{wall}+R_{cold}+R_{hot}$$

Among them, the total thermal resistance *R_tot_* can be calculated based on the Logarithmic Mean Temperature Difference (LMTD), the total heat transfer area n*S* of the plate heat exchanger, and the heat transfer rate *Q*:

$$R_{tot}=\frac{LMTDnS}{Q}$$

Among them, the logarithmic mean temperature difference is calculated from *ΔT*_min_ and *ΔT*_max_ among the temperature differences of the end plates of the plate heat exchanger:

$$LMTD\text{=}\frac{\Delta T_{\max}-\Delta T_{\min}}{\ln\frac{\Delta T_{\max}}{\Delta T_{\min}}}$$

Among them, the heat transfer rate *Q* is the average value of the heat transfer rates on the cold and hot sides of the plate heat exchanger:

$$Q=\frac{c_{p,cold}q_{m,cold}\Delta T_{cold}+c_{p,hot}q_{m,hot}\Delta T_{hot}}{2}$$

Among them, *c_p_* is the specific heat capacity of the working fluid, *q_m_* is the mass flow rate, and ΔT is the temperature difference on one side. The thermal conduction resistance *R_wall_* of the plate is only related to the thickness *δ*, thermal conductivity *λ*, and the number of plates of the heat exchange plate. The single-phase heat transfer Nusselt number correlation of the plate heat exchanger can be represented by a mathematical model in the following form:

$$Nu=CRe^{N}Pr^{1/3}(\frac{\mu_{l}}{\mu_{wall}})^{0.14}$$

Among them, C and N are undetermined coefficients, *(μ_l_/μ_wall_)^0.14^* is the correction term,*μ_l_* is the dynamic viscosity corresponding to the mainstream temperature *T_l_* of the fluid, and *μ_wall_* is the dynamic viscosity corresponding to the wall temperature *T_wall_.*

To solve the single-phase heat transfer correlation of the plate heat exchanger, the undetermined coefficients C and N need to be determined. First, an initial value is set for the Reynolds number exponent N, and the parameter C corresponding to the currently set exponent N can be obtained by fitting the experimental data using the least square method. After each round of calculation, a check calculation of the exponent N is required until the error meets the allowable range, and finally C and N are obtained.
